# Th1-Like ICOS^+^ Foxp3^+^ T_reg_ Cells Preferentially Express CXCR3 and Home to β-Islets during Pre-Diabetes in BDC2.5 NOD Mice

**DOI:** 10.1371/journal.pone.0126311

**Published:** 2015-05-06

**Authors:** Mara Kornete, Edward S. Mason, Julien Girouard, Erin I. Lafferty, Salman Qureshi, Ciriaco A. Piccirillo

**Affiliations:** 1 Department of Microbiology and Immunology, McGill University, Montréal, QC, Canada, H3A 2B4; 2 Department of Medicine, McGill University, Montreal, QC, Canada; 3 Microbiome and Disease Tolerance Centre, McGill University, Montreal, QC, Canada, H3A 2B4; 4 FOCIS Center of Excellence in Translational Immunology, Research Institute of the McGill University Health Centre, Montreal, QC, Canada, H3G 1A4; 5 Division of Experimental Medicine, McGill University, Montreal, QC, Canada

## Abstract

Type 1 diabetes (T1D) occurs through a breakdown of self-tolerance resulting in the autoimmune destruction of the insulin producing β-islets of the pancreas. A numerical and functional waning of CD4^+^Foxp3^+^ regulatory T (T_reg_) cells, prompted by a pancreatic IL-2 deficiency, accompanies Th1 autoimmunity and T1D progression in non-obese diabetic (NOD) mice. Recently, we identified a dominant subset of intra-islet T_reg_ cells that expresses the ICOS costimulatory receptor and promotes self-tolerance delaying the onset of T1D. ICOS co-stimulation potently enhances IL-2 induced survival and proliferation, and suppressive activity of T_reg_ cells *in situ*. Here, we propose an ICOS-dependent mechanism of T_reg_ cell homing to the β-islets during pre-diabetes in the NOD model via upregulation of the CXCR3 chemokine receptor. The islet-specific ICOS^+^ T_reg_ cell subset preferentially expresses CXCR3 in the pancreatic lymph nodes (pLN) in response to T_eff_ cell-mediated pancreatic inflammation, an expression correlating with the onset and magnitude of IFN-γ production by T_eff_ cells in pancreatic sites. We also reveal that intra-pancreatic APC populations and insulin-producing β, but not α nor δ, islet cells secrete the CXCR3 chemokines, CXCL9, 10 and 11, and selectively promote ICOS^+^CXCR3^+^ T_reg_ cell chemotaxis *in vitro*. Strikingly, islet-derived T_reg_ cells also produce these chemokines suggesting an auto-regulation of homing by this subset. Unlike ICOS^-^ cells, ICOS^+^ T_reg_ cells adopt a Th1-like T_reg_ phenotype while maintaining their suppressive capacity, characterized by expression of T-bet and CXCR3 and production of IFN-γ in the draining pLNs. Finally, *in vivo* neutralization of IFN-γ blocked T_reg_ cell CXCR3 upregulation evincing its role in regulating expression of this chemokine receptor by T_reg_ cells. Thus, CXCR3-mediated trafficking of T_reg_ cells could represent a mechanism of homeostatic immunoregulation during diabetogeneesis.

## Introduction

Mechanisms of peripheral immune self-tolerance prevent the onset and progression of pathological autoimmune responses. Immunosuppressive CD4^+^Foxp3^+^ T regulatory (T_reg_) cells, constitutively expressing CD25 (IL-2Rα), develop in the thymus (tT_reg_) or differentiate from non-regulatory CD4^+^Foxp3^-^ T effector (T_eff_) cells *in vitro* (iT_reg_) or in the periphery (pT_reg_) [1], [2]. In order to establish and maintain dominant self-tolerance, T_reg_ cells employ a plethora of immunosuppressive mechanisms including production of anti-inflammatory cytokines like TGF-β and IL-10, thereby inhibiting T_eff_ cell expansion and effector functions. Developmental blockade of this lineage in mice via day 3 thymectomy provokes lympho-proliferative and multi-organ autoimmune disease [1]. Similarly, loss-of-function mutations in the T_reg_ cell lineage-specifying transcription factor Foxp3 abrogate T_reg_ cell development, resulting in severe autoimmunity in *scurfy* mice and immunodysregulation polyendocrinopathy enteropathy X-linked syndrome (IPEX) in humans [3].

NOD mice succumb to autoimmune diabetes resulting from a T-cell dependent destruction of the insulin producing β-islets of Langerhans [4]. Diabetogenesis in the NOD model shares many features with human T1D including insulin-responsive hyperglycemia, common risk loci, and the development of pancreas-specific auto-antibodies [4]. Inflammatory infiltrates are observed in the islets at 3–4 weeks of age however outright insulitis does not occur until 4–8 weeks later, suggesting immunoregulatory mechanisms are at least partially intact during this period. BDC2.5-NOD mice carry a transgenic TCR specific to a β-islet antigen, facilitating reliable, synchronous diabetes onset and transfer of diabetes via infusion of cells into lymphopenic hosts. Progression to insulitis in NOD and BDC2.5 mice results from the failure of multiple central and peripheral immune checkpoints. This includes a progressive waning in function and number of intra-islet T_reg_ cell populations [5, 6, 7]. Recently, we and others have implicated an islet-specific deficiency in IL-2, a cytokine critical for T_reg_ cell homeostasis, in the functional waning of T_reg_ cells at the onset of insulitis [8, 9, 10]. Conversely, low dose IL-2 therapy both maintains pancreatic T_reg_ cell populations and protects NOD mice from T1D [9, 7]. In addition, we recently demonstrated a critical dependence on the ICOS co-stimulatory pathway for the survival and function of intra-pancreatic T_reg_ cells [7]. Specifically, the ICOS^+^ T_reg_ cell subset, predominates in the islets of pre-diabetic NOD mice, and preferentially survives, proliferates and suppresses effector responses *in situ*. A decline in ICOS expression by T_reg_ cells accompanies the progression to insulitis whereas IL-2 therapy selectively maintains the intra-islet ICOS^+^ T_reg_ cell pool and protects from T1D. Furthermore, co-transfer of ICOS^+^ T_reg_ cells, but not their ICOS^-^ or *icos* deficient (ICOS^-/-^) counterparts, protects lymphopenic NOD recipients from T_eff_ cell-induced insulitis and disease. Thus, a local network of cytokine and co-stimulatory mechanisms can co-ordinate T_reg_ cell homeostasis in the pre-diabetic islets of autoimmune-prone mice.

While ICOS^+^ T_reg_ cells preferentially accumulate in the pre-diabetic islets, the distinct mechanisms that mediate homing are unclear. Thus, we hypothesized that ICOS could also play a role in their preferential homing and accumulation of T_reg_ cells in pancreatic sites of BDC2.5 TCR transgenic NOD mice. Recent studies have identified T_reg_ sub-populations that express T_eff_ cell-associated genes critical for their trafficking to sites of inflammation [11]. Notably, IFN-γ was shown to induce T-bet and CXCR3 expression in T_reg_ cells, thus promoting their homing to sites of type-1 inflammation [12]. Similarly, IRF4 and STAT3 expression by T_reg_ cells is necessary for suppression of pathogenic Th2 and Th17 responses, respectively [13, 14]. Given that ICOS^+^ T_reg_ cells represent a dominant, suppressive subset that accumulates in a Th1-polarized environment, we hypothesized that ICOS^+^ T_reg_ cells preferentially home to the inflamed islets using CXCR3.

Here, we show that ICOS^+^ T_reg_ cells preferentially express CXCR3 in pLN of pre-diabetic NOD mice, a process dependent on expression of ICOS. IFN-γ produced by islet-reactive T_eff_ cells *in vivo* induces expression of CXCR3 by ICOS^+^ T_reg_ cells and correlates with the onset and magnitude of inflammation in pancreatic sites. Furthermore, unlike ICOS^-^ cells, CXCR3-expressing ICOS^+^ T_reg_ cell from pLN adopt a Th1-like phenotype, characterized by production of IFN-γ, and expression T-bet and IFN-γR, and preferentially respond to IFN-γ *in vitro* through activation of STAT1. Moreover, CXCR3 chemokines are specifically expressed by intra-pancreatic macrophages and dendritic cells, and surprisingly by insulin-producing β but not α or δ islet cells. Intriguingly, intra-pancreatic T_reg_ cells were also found to express CXCR3 ligands. Finally, *in vivo* neutralization of IFN-γ significantly blunted CXCR3 upregulation by ICOS^+^ T_reg_ cells. Collectively, this data indicates trafficking of T_reg_ cells represents a mechanism of homeostatic regulation of autoimmunity in T1D.

## Materials and Methods

### Mice

NOD.TCRα^-/-^ mice were a generous gift from C. Benoist (Harvard University). BDC2.5.Foxp3^GFP^ ICOS^-/-^, BDC2.5.Foxp3^GFP^ and BDC2.5. Foxp3^GFP^ Thy1.1 mice were generated in house. All mouse strains were maintained in SPF conditions at RI-MUHC.

### Antibodies and flow cytometry

Single cell suspension prepared from different organs were stained and acquired on FACSCanto (BD) or BD Fortessa and analyzed with FlowJo software (Tree Star). Surface phenotype staining was done with the following fluorochrome-conjugated or biotinylated mAbs: anti-CD3 (145-2c11), anti-CD4 (RM5), anti-ICOS (7E.17G9), anti-Thy1.1 (HIS51), anti-CD11c (HL3), anti-F4/80 (BM8), anti-CXCR3 (cxcr3-173), and anti-IFN-γR (2E2). The expression of Foxp3 (FJK-16s), T-bet (ebio4B10), CXCL9 (MIG-2F5.5) (eBioscience, San Diego, CA), CXCL10 (CL9177B) (Cedarlane, Burlington, ON), CXCL11 (rmCXCL11) (R&D systems, Burlington, ON), Ki-67 (B56) (BD Bioscience, Mississauga, Ontario) was determined by intracellular staining performed according to manufacturers’ protocols. The STAT1 phosphorylation assay was done following BD recommendations. To evaluate cytokine production, T cells were re-stimulated for 4hrs at 37°C with PMA (20ng/ml), ionomycin (1nM) (Sigma-Aldrich, Oakville, Canada) and BD GolgiStop^TM^ (1:1000 dilution), and then stained intracellularly with anti-IFN-γ (XMG1.2) (eBioscience, San Diego, CA). T-bet fluorescence minus one (FMO) control and antibody titration is displayed in S1E Fig.

### Cell purification

For some *in vivo* adoptive transfer studies, T_reg_ and T_eff_ cells from respective mouse strains were purified from splenocytes and LN cells either based on CD4 and CD25 expression using the autoMACS cell sorter, using BDC2.5.Foxp3^GFP^ reporter mice (Miltenyi Biotech, San Diego, CA). Specifically, CD4^+^CD25^+^ (>90% purity) and CD4^+^CD25^−^ (>93% purity) T cell fractions were obtained by positive selection. For some *in vivo* adoptive transfer studies and *in vivo* suppression assays, T cell subsets were isolated using a FACSAria Cell Sorter with a purity >98%. CD4^+^Foxp3^+^ T_reg_, CD4^+^Foxp3^-^ T_eff_ or CXCR3^+^ or CXCR3^-^ ICOS^+^ T_reg_ cells were sorted from BDC2.5Foxp3^GFP^ reporter mice.

### Adoptive T cell transfers

MACS purified CD4^+^CD25^-^ (T_eff_,) or CD4^+^CD25^+^ (T_reg_) T cells (7.5X10^5^ cells) from BDC2.5Foxp3^GFP^ donor mice were transferred intravenously (i.v.) into NOD.TCRα^-/-^ recipient mice at the indicated ratios. In some instances, T cell populations were FACS-purified from the above-described mouse strains. Blood glucose levels were determined every 2–3 days with Hemoglucotest kits (Roche Diagnostics, Montreal, Canada) and T1D was diagnosed at values >300 mg/dl.

### *In vivo* antibody treatment

NOD.TCRα^-/-^ mice were reconstituted with MACS-sorted CD4^+^CD25^+^ (T_reg_) or CD4^+^CD25^-^ (T_eff_) cells either alone or at the indicated ratios. Mice also received i.p injections of either vehicle (PBS) or a neutralizing IFN-γ mAb (clone XMG1.2, 1.6 mg per kg), one day before transfer and every two days post-adoptive transfer, for a total of 18 days.

### ELISA

DuoSet ELISA kits (R&D Systems) were used to analyze IFN-γ (DY485) and CXCL10 (DY466) in whole-pancreas protein samples.

### Migration assay

CD11c^+^ dendritic cells and F4/80^+^ macrophages from NOD.TCRα^-/-^ mice and CD4^+^ cells from BDC2.5 mice were isolated via MACS. Cells were resuspended in GIBCO RPMI media (10% FBS) and incubated at 2 x 10^6^ cells/ml. CD11c^+^ cells and F4/80^+^ cells were plated at 10^6^ cells/well in the bottom compartments of a 24-well Transwell with 5.μm pore polycarbonate membrane (Corning). CD4^+^ T cells were plated in the top of the wells at 10^5^ cells/well and allowed to migrate for 3 hours at 37°C. Migrated cells were assessed via flow cytometry as described above.

### Statistical analysis

Results are expressed as means ± SD. Analyses were performed with a Student’s *t* test, values of *p* < 0.05 were considered significant. For p values: * = <.05, ** = <.01, *** = <.001

## Results

### ICOS-dependent expression of CXCR3 by ICOS^+^ T_reg_ cells in the pancreatic LN

We previously showed that ICOS^+^ T_reg_ cells, compared to ICOS^-^ T_reg_ cells, display an increased functionally fit phenotype and preferentially accumulate in pre-diabetic islets of BDC2.5 NOD mice [7]. *In vivo* ICOS blockade induces changes in the gene expression profile of intra-pancreatic T_reg_ cells, including genes encoding various chemokine receptors, such as CCR2, CCR5 and CXCR3 [15]. This prompted us to examine whether specific chemokine receptors, regulated in part by inflammation, could promote ICOS^+^ T_reg_ trafficking to the β-islets.

**Fig 1:**
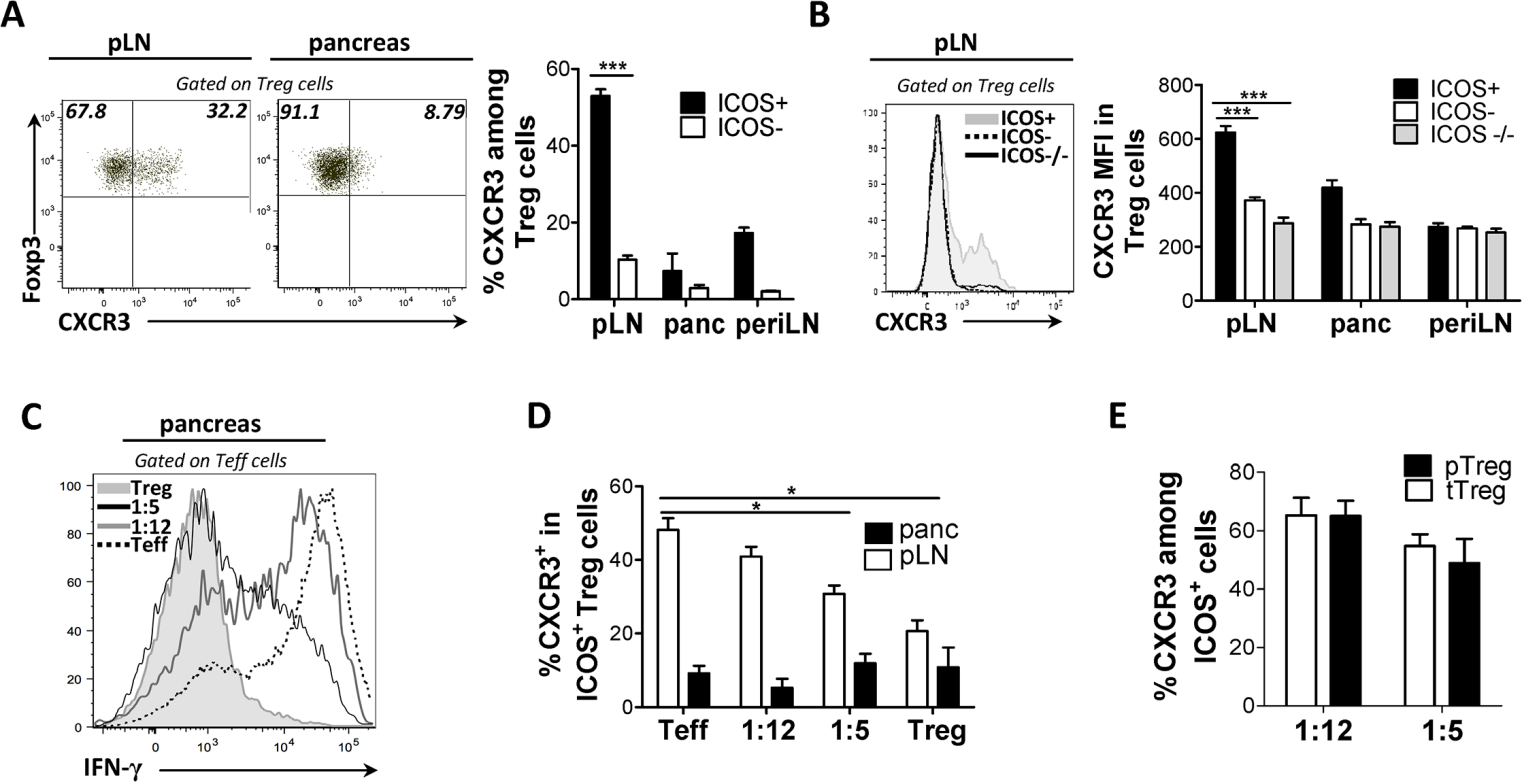
Autoreactive T cell-mediated inflammation induces CXCR3 expression by ICOS^+^ T_reg_ cells prior to T1D onset. Cell suspensions of pancreas and draining LN from 4 week old (A, B) WT and (B) ICOS^**-/-**^ BDC2.5 mice were assessed for the frequency of CXCR3^**+**^ cells and levels of CXCR3 expression (MFI) between the ICOS^**+**^ and ICOS^**-**^ subsets of T_**reg**_ cells. (C and D) NOD.TCRα^**-/-**^ mice received MACS sorted BDC2.5 CD4^**+**^CD25^**+**^ (T_**reg**_, 0.75X10^**5**^) or CD4^**+**^CD25^**-**^ (T_**eff**_, 7.5X10^**5**^) cells alone or at the indicated T_**reg/**_T_**eff**_ cell ratios. When the T_**eff**_ cell recipient mice displayed hyperglycemia (>33mmol/L), mice were sacrificed and expression of IFN-γ by T_**eff**_ cells (C) and CXCR3^**+**^ percent cells among the ICOS^**+**^ T_**reg**_ cell subset were assessed. (E) NOD.TCRα^**-/-**^ mice received the indicated ratios of FACS-sorted Thy1.2^**+**^ T_**reg**_ cells to 7.5X10^**5**^ BDC2.5 Thy1.1^**+**^ CD4^**+**^Foxp3^**-**^ T_**eff**_ cells. After 14 days, the Thy1.2 (tT_**reg**_) and Thy1.1^**+**^ (pT_**reg**_) subsets of ICOS^**+**^ T_**reg**_ cells were compared for percent CXCR3^**+**^ cells. (n = 4, peri LN = pooled axial, brachial and inguinal peripheral lymph nodes).

We initially screened ICOS^+^ or ICOS^-^ T_reg_ cells for expression of chemokine receptors including CXCR3, CCR2, CCR7, CCR4, CCR9, CXCR5 and CCR6 (data not shown). We found that in contrast to other T cell subsets, ICOS^+^ T_reg_ cells displayed marked CXCR3 expression in the draining pLNs of pre-diabetic BDC2.5 NOD mice. Specifically, CXCR3 was highly expressed by ICOS^+^ T_reg_ cells, relative to ICOS^-^ T_reg_ cells in terms of both frequency (52.9% vs. 10.31%) and level of expression (MFI 624 vs. 371), in the draining pLN but not in other lymphoid tissues (Fig 1A). Though ICOS^+^ T_reg_ cells did not express CXCR3 in the pancreas, CXCR3 is known to undergo rapid internalization and degradation upon encounter with its ligands at the site of inflammation [16]. Moreover, T_reg_ cells from BDC2.5 ICOS^-/-^ mice expressed low CXCR3 at all sites examined, indicating that CXCR3 expression in T_reg_ cells is regulated by ICOS (Fig 1B). Unexpectedly, only a low fraction T_eff_ cells expressed CXCR3 in BDC2.5 mice in all sites examined (S1A Fig). Overall, ICOS-dependent CXCR3 expression coincided with specific accumulation of ICOS^+^ T_reg_ cells within the target organ, suggesting this subset may use CXCR3 to home to pancreatic sites.

### Autoreactive T cell-mediated inflammation induces CXCR3 expression by ICOS^+^ T_reg_ cells prior to T1D onset

Pro-inflammatory cytokines modulate expression of chemokine receptors to induce immune infiltration of the inflammatory lesion. Thus, we hypothesized that expression of CXCR3 is induced on T_reg_ cells in response to intra-pancreatic inflammatory events. Most notably, intra-islet IFN-γ production, primarily by T_eff_ cells, was of particular interest considering its various inflammatory actions on immune and islet cells in NOD mice. To determine if inflammation influenced ICOS^+^ T_reg_ cell homing to pancreatic sites, we reconstituted NOD.TCRα^-/-^ mice with CD4^+^CD25^-^ T_eff_ (almost exclusively GFP^-^) or CD4^+^CD25^+^ T_reg_ cells from BDC2.5.Foxp3^GFP^ reporter mice either alone or at different T_eff_/T_reg_ cell ratios to mimic different inflammation levels. The transfer of BDC2.5 T cells in NOD.TCRα^-/-^ mice reliably and predictably induces different levels of pancreatic inflammation, and allows for the tracking of T cell functional dynamics at T1D onset and progression. As expected, mice receiving only T_eff_ cells succumbed to rapid T1D onset by day 12 (data not shown), and this correlated with increased production of IFN-γ by auto-reactive T_eff_ cells in the pLN (S1B Fig) and pancreas (Fig 1C). Interestingly, CXCR3 was readily expressed on ICOS^+^ T_reg_ cells in all mice co-transferred with T_reg_ cells. The frequency of CXCR3^+^ T_reg_ cells declined with decreased T_eff_ cell-induced inflammation suggesting that pancreatic inflammation promotes the induction of CXCR3 on T_reg_ cells (40% at 1:12 ratio, 30% at 1:5 ratios, and 21% with T_reg_ cells alone) (Fig 1D). Intriguingly, our results show that mice reconstituted with T_eff_ cells alone contained the highest frequency of CXCR3^+^ T_reg_ cells in the pLN on day 12 (49%) suggesting that CXCR3 can be readily upregulated amongst pT_reg_ cell populations induced from transferred T_eff_ cells, very few of which express CXCR3 (Fig 1D). In order to directly determine whether CXCR3 is differentially expressed by tT_reg_ or pT_reg_ cells, we assessed CXCR3 expression relative to Foxp3 expression in transferred FACS sorted Thy 1.1^+^ (T_eff_ GFP^-^)/Thy 1.2^+^ (tT_reg_ GFP^+^) congenic cells. We found both pools of T_reg_ cells were equally capable of expressing CXCR3 (Fig 1E), demonstrating that tT_reg_ or pT_reg_ cells upregulate CXCR3, in contrast to T_eff_ cells, as pancreatic inflammation increases.

### CXCR3 expression defines a functionally fit subpopulation of ICOS^+^ T_reg_ cells

**Fig 2:**
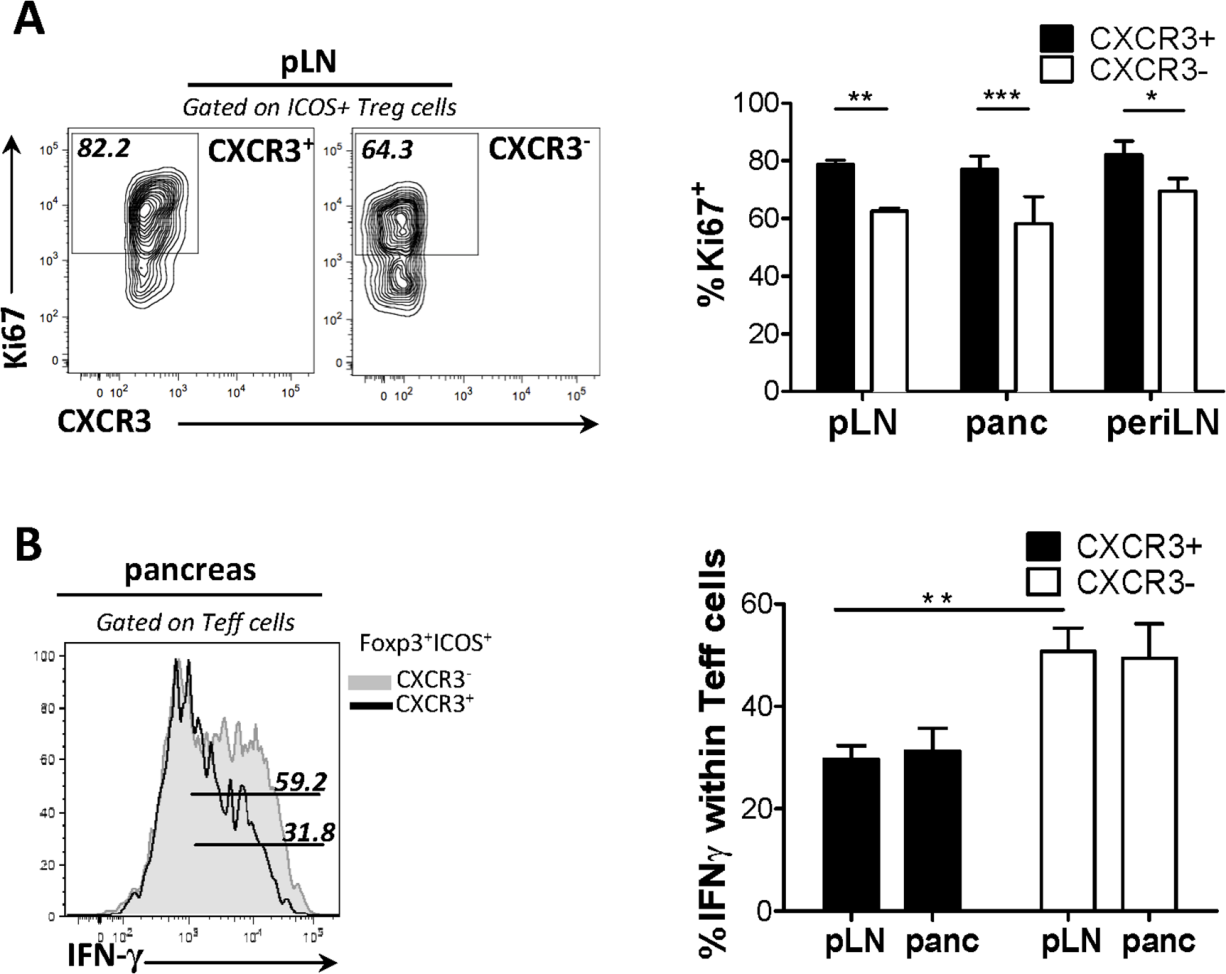
CXCR3 expression delineates a functionally fit subpopulation of ICOS^+^ T_reg_ cells. (A) Cell suspensions of pancreatic draining LN from 4-week-old mice were isolated and percent cycle cells, determined by Ki-67 expression, were compared between the CXCR3^**+**^ and CXCR3^**-**^ subsets of ICOS^**+**^ T_**reg**_ cells. (B) NOD.TCRα^**-/-**^ mice received T_**eff**_ (7.5X10^**5**^) cells and either CXCR3^**+**^ or CXCR3^**-**^ ICOS^**+**^ T_**reg**_ cells (7.5X10^**4**^) cells isolated from pooled LN and spleen of BDC2.5 mice. After 14 days suppression was assessed via percent IFN-γ^**+**^ cells among T_**eff**_ cells in the pancreas and draining LN. (n = 4).

Next, we examined the phenotype and suppressive function of CXCR3^+^ICOS^+^ T_reg_ cells, compared to their CXCR3^-^ counterparts. In all sites examined, CXCR3^+^ T_reg_ cells were more proliferative than CXCR3^-^ T_reg_ cells, as indicated by the frequency of Ki-67 positive cells (Fig 2A). In order to determine the relative suppressive capacity of CXCR3^+^ ICOS^+^ T_reg_ cells in our T1D transfer model, we reconstituted NOD.TCRα^-/-^ mice with FACS-sorted T_eff_ cells alone or co-transferred with either CXCR3^+^ICOS^+^ T_reg_ cells or CXCR3^-^ICOS^+^ T_reg_ cells. We observed that CXCR3^+^ICOS^+^ T_reg_ cells were endowed with a more potent suppressive potential *in situ* than CXCR3^-^ ICOS^+^ T_reg_ cells, as the former suppressed IFN-γ production by T_eff_ cells, in the pLN and pancreas more efficiently (Fig 2B). Thus, CXCR3-expressing ICOS^+^ T_reg_ cells represent a functionally fit subset with potent suppressive activity *in situ*.

### CXCR3-expressing ICOS^+^ T_reg_ cells adopt a Th1-like phenotype in the pancreatic draining lymph nodes

**Fig 3:**
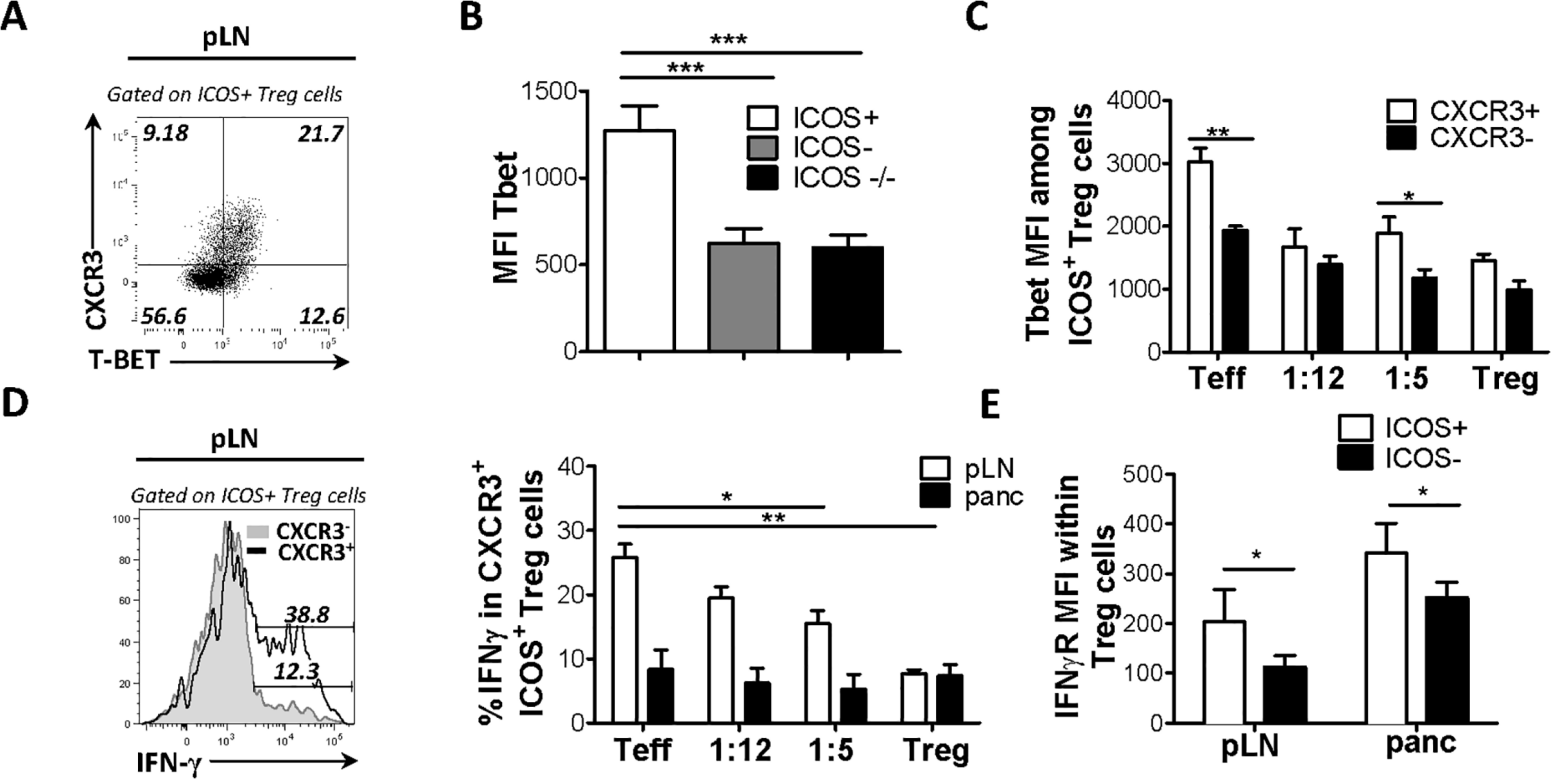
CXCR3-expressing ICOS^+^ T_reg_ cells adopt a Th1-like phenotype in draining LN. T-bet and CXCR3 co-expression was assessed in ICOS^**+**^ T_**reg**_ cells from 4 week old WT BDC2.5 mice (A). T-bet expression (MFI) between ICOS^**+**^ and ICOS^**-**^ subsets within T_**reg**_ cells (B). NOD.TCRα^**-/-**^ mice received BDC2.5 CD4^**+**^CD25^**+**^ T_**reg**_ or CD4^**+**^CD25^**-**^ T_**eff**_ cells (0.75X10^**5**^) at the indicated T_**reg/**_T_**eff**_ cell ratios. When mice receiving T_**eff**_ cells alone became hyperglycemic (>33mmol/L), mice were sacrificed and the frequency of (C) T-bet^**+**^ and (D) IFN-γ^**+**^ cells within the ICOS^**+**^ T_**reg**_ subset was assessed. (E) NOD.TCRα^**-/-**^ mice received WT T_**eff**_ (7.5X10^**5**^) cells and ICOS^**-/+**^ T_**reg**_ (7.5X10^**5**^) cells isolated from pooled LN and spleen. 10 days post-transfer, cell suspensions were obtained from the pancreas and draining pLN and IFN-γR expression (MFI) was compared between ICOS^**+**^ and ICOS^**-**^ T_**reg**_ cells. (3B-3D n = 4, 3E n = 5)

We investigated whether ICOS^+^ T_reg_ cells expressed markers that would functionally specify them for suppression at sites of type 1 inflammation. Notably, expression of CXCR3 by T_reg_ cells depends on co-expression of Foxp3 and the Th1 lineage specifying transcription factor T-bet [12]. Thus, we predicted T-bet would be co-expressed with CXCR3 by ICOS^+^ T_reg_ cells. We observed a marked co-expression of CXCR3 and T-bet by *ex vivo* T_reg_ cells in pLN of BDC2.5 NOD mice (Fig 3A). Moreover, robust T-bet expression was confined to the ICOS^+^ subset of T_reg_ cells in the pLN of BDC2.5 mice and was significantly reduced in ICOS^-/-^ T_reg_ cells suggesting that T-bet expression is partly ICOS-dependent (Fig 3B). Concordant with CXCR3 expression, there was also a trend of a positive correlation between T-bet expression in ICOS^+^ pT_reg_/tT_reg_ cells and IFN-γ, which was mostly produced by pancreatic Teff cells, in the adoptive transfer model (Fig 3C and Fig 1C). Strikingly, following adoptive transfer of T1D, BDC2.5 CXCR3^+^ ICOS^+^ T_reg_ cells expressed IFN-γ in the pLN, despite their enhanced suppressive potential in the pancreas. The frequency of IFN-γ-expressing T_reg_ cells in the pLN correlated with the degree of pancreatic inflammation (Fig 3D). Furthermore, we show that ICOS^+^ T_reg_ cells express IFN-γR at higher levels than ICOS^-^ T_reg_ cells at each site examined, consistent with a Th1-like phenotype (Fig 3E). Thus, unlike ICOS^-^ T_reg_ subset, ICOS^+^ T_reg_ cells adopt a Th1-like phenotype in the draining pancreatic LN that correlates with Th1-polarized inflammation in the pancreas.

### Pancreatic antigen presenting cells produce CXCR3 ligands

**Fig 4:**
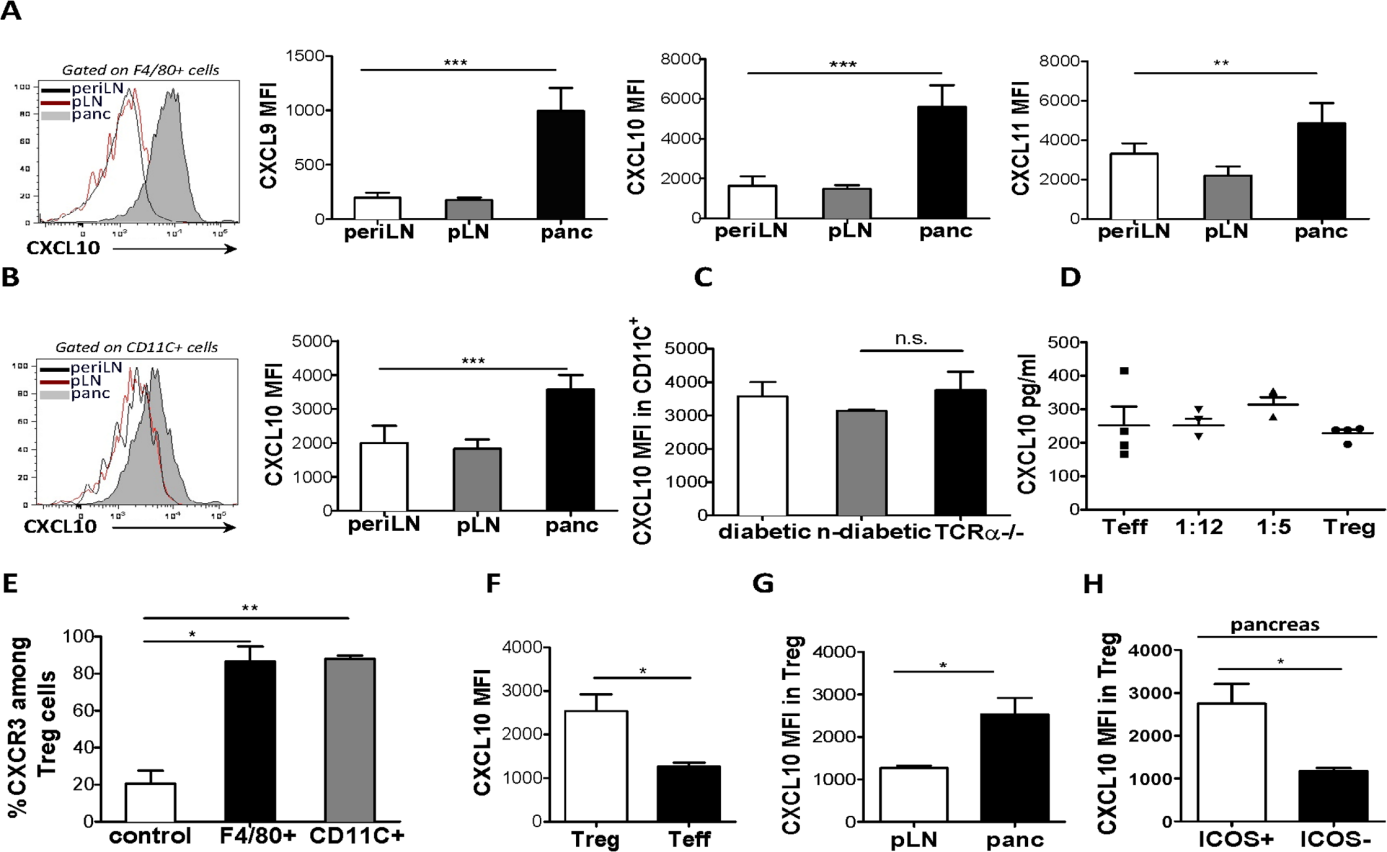
Resident leukocyte subsets in the pancreas express CXCR3-activating chemokines. NOD.TCRα^**-/-**^ mice initially received BDC2.5 CD4^**+**^ T cell (7.5X10^**5**^) cells, and then cell suspensions from pancreas, draining pLN and peripheral LN of diabetic mice were examined for expression of CXCL9, CXCL10 and CXCL11 in F4/80^**+**^ (A) and CXCL10 in CD11c^**+**^ (B) cells was assessed by flow cytometry. (C) NOD.TCRα^**-/-**^ mice were transferred with CD4^**+**^ T cells in order to induce T1D. Following adoptive transfer, glucose levels were measured daily in order to assess diabetes onset. Pancreatic cell suspensions from recipients were obtained and CXCL10 expression (MFI) was compared among CD11c^**+**^ cells from diabetic or non-diabetic recipients, as well as un-transferred (without T cell transfer) NOD.TCRα^**-/-**^ mice. (D) NOD.TCRα^**-/-**^ mice received T_**reg**_ or T_**eff**_ cells either alone or at the indicated T_**reg/**_T_**eff**_ cell ratios. When mice receiving T_**eff**_ cells alone became hyperglycemic (>33mmol/L), CXCL10 levels were assessed by ELISA in supernatants from pancreatic suspensions. (E), F4/80^**+**^ and CD11c^**+**^ (1X10^**6**^/well) cells were seeded in triplicate in lower chambers, and CD4^**+**^ (1X10^**6**^/well) cells in the upper chambers of 24 well Transwell plate. Following a 3 hour incubation period, the percent migrated CXCR3^**+**^ cells among ICOS^**+**^ T_**reg**_ cells was compared between wells containing APCs and media alone (*control*). Cell suspensions of pancreas and draining LN of 4-week-old BDC2.5 mice were obtained and CXCL10 expression (MFI) was compared (F) between pancreatic T_**reg**_ cells and T_**eff**_ cells (G), between T_**rec**_ cells at sites indicated, and between ICOS^**+**^ and ICOS^**+**^ T_**reg**_ cells within pancreas (H). (4A-4C n = 6, 4D n = 4, 4E n = 2, 4F-4H n = 5).

We then examined the expression of the three CXCR3-activating chemokines, CXCL9, CXCL10 and CXCL11, in the pancreas, which we hypothesized could account for the influx of ICOS^+^ T_reg_ cells from the pLN. Furthermore, as IFN-γ has been shown to induce expression of these chemokines in numerous settings [17, 18], we predicted that chemokine expression should increase with pancreatic inflammation, along with CXCR3. In 4-week-old BDC2.5 mice, we observed expression of CXCL9, CXCL10, and CXCL11 (Fig 4A and 4B; data not shown), by APC populations resident to the pancreas, specifically F4/80^+^ macrophages (Fig 4A) and CD11c^+^ DCs (Fig 4B). This level of expression was not observed by lymphoid tissue-derived APC subsets (Fig 4A and 4B; data not shown). To determine whether APC chemokine production could induce specific T_reg_ homing, Transwell assays were performed. To this end, MACS-sorted F4/80^+^ macrophages or CD11c^+^ DC cells were seeded in the lower chamber while CD4^+^ T cells were seeded in the upper chamber. The number of migrating cell types in the lower chamber was then evaluated following a 3-hour incubation period. CXCR3^+^ T_reg_ cells were markedly enriched in the bottom chamber that contained APCs but not those containing media alone (Fig 4E). Thus, CXCR3^+^ T_reg_ cells specifically migrated towards the CXCR3 chemokine-producing APC populations *in vitro*.

In order to examine the effects of different levels of inflammation on chemokine expression NOD.TCRα^-/-^ mice either received CD4^+^ T cells in order to induce T1D (Fig 4C) or the adoptive transfer experiments were performed as described in Fig 1 (Fig 4D). Unexpectedly, chemokine expression by APCs did not increase in the presence of BDC2.5 T cell-mediated inflammation in NOD.TCRα^-/-^ recipients, irrespective of T1D outcome (Fig 4C and S1C Fig). Furthermore, we found no change in CXCL10 concentrations in pancreatic supernatants or frequency of CXCL9/10 or 11^+^APCs in the presence of BDC2.5 T cell-mediated inflammation in NOD.TCRα^-/-^ recipients, irrespective of the transferred BDC2.5 T_eff_ /T_reg_ cell ratio (Fig 4D and data not shown)_._ Thus, APC expression of CXCL9, CXCL10 and CXCL11 was confined to the pancreas of BDC2.5 NOD mice but not upregulated in response to inflammation.

### Intra-pancreatic Foxp3^+^T_reg_ cells express CXCR3 chemokines

Recent evidence indicates that in addition to a range of cytokines, T_reg_ cells can also produce chemokines in certain inflammatory contexts [19]. Thus, we predicted that pancreatic T_reg_ cells upregulate CXCR3 chemokines to augment recruitment of T_reg_ cells to the inflammatory lesion. We found that T_reg_ cells in the pancreas, compared to T_eff_ cells, expressed significantly higher levels of CXCL9, CXCL10 and CXCL11 (Fig 4F and data not shown). This was specific to pancreatic T_reg_ cells, as T_reg_ cells from other sites did not express as high levels of the ligands (Fig 4G). Intriguingly, the ICOS^+^ subset expressed significantly higher levels of CXCR3 ligands than ICOS^-^ T_reg_ cells (Fig 4H). Thus, we observed chemokine expression by Foxp3^+^ T_reg_ cells, which could represent a mechanism of intra-subset regulation of homing into inflamed target organs.

### Intra-islet T_reg_ cells reverse T_eff_ cell-mediated abrogation of chemokine secretion by ß-islet cells

**Fig 5:**
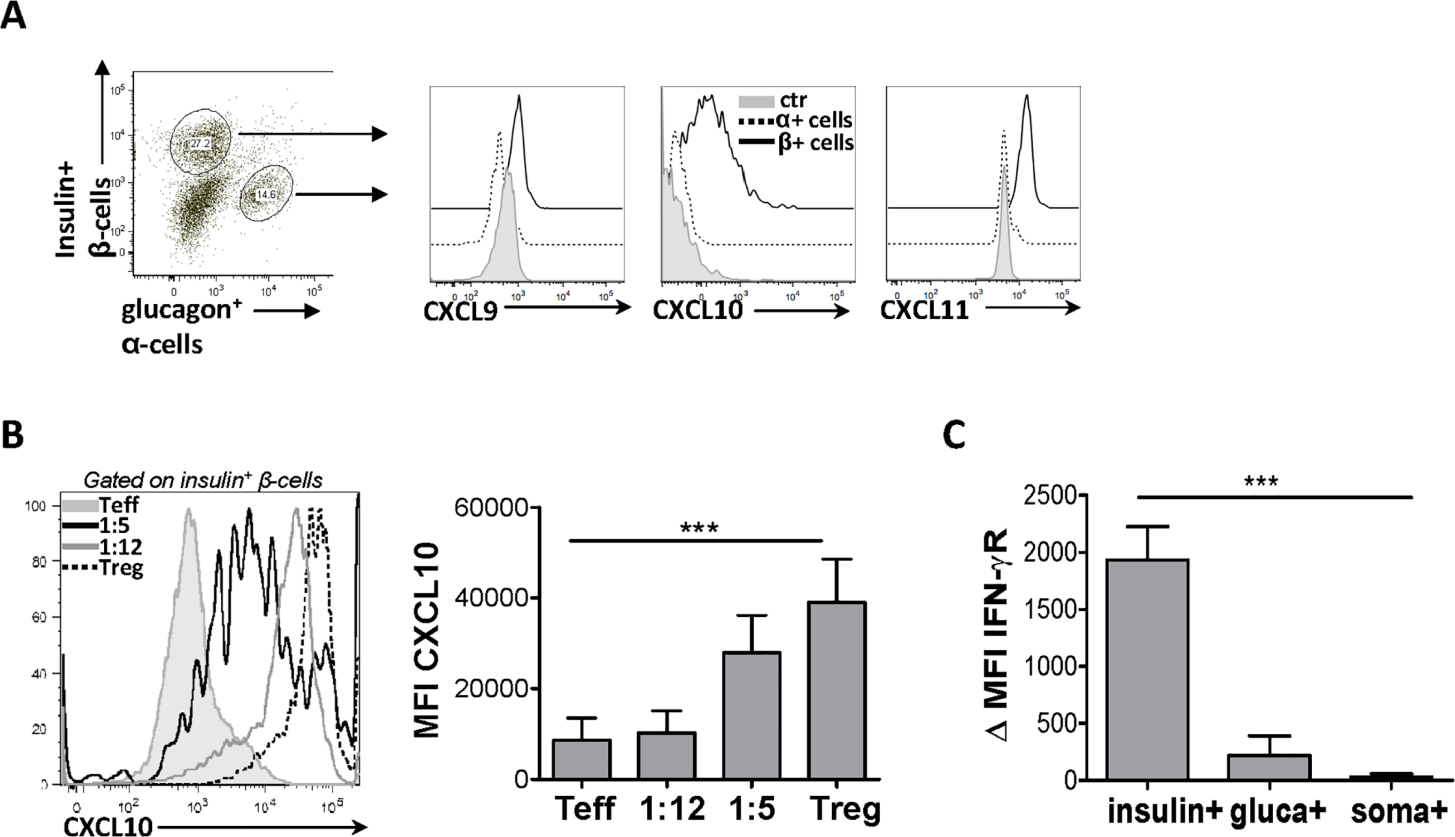
Intra-islet T_reg_ cells reverse T_eff_ cell-mediated abrogation of chemokine secretion by ß-islet cells. β-islet cells were isolated from 4-week-old BDC2.5 mice. (A) Expression of CXCR3 chemokines was compared between insulin^**+**^ cells and glucagon^**+**^ cells, and unstained controls. (B), NOD.TCRα^**-/-**^ mice received T_**reg**_ or T_**eff**_ cells either alone or at the indicated T_**reg/**_T_**eff**_ cell ratios. When mice receiving T_**eff**_ cells alone became hyperglycemic (>33mmol/L), CXCL10 expression (MFI) in β-islet cells was compared between groups. (C) IFN-γR ΔMFI (ΔMFI was calculated by subtracting the isotype control MFI from IFN-γR antibody MFI) and was compared between β (insulin^**+**^), α (glucagon^**+**^) and δ (somatostatin^**+**^) cells isolated from 4-week-old BDC2.5 mice. (5B, 5C n = 5).

It has recently been established that endocrine β-cells regulate various immune cell subsets during NOD mouse diabetes progression [20]. Therefore, we sought to determine whether endocrine cells in BDC2.5 mice could express CXCR3 chemokines and contribute to the recruitment of T_reg_ cells to pancreatic sites. Using our method recently developed to assess the phenotype and function of pancreatic islet cell populations by flow cytometry [21] we observed substantial expression of CXCL9, CXCL10 and CXCL11 specifically by insulin^+^ β-cells compared to the glucagon^+^ α-cells (Fig 5A) and somatostatin^+^ δ-cells (data not shown). While all three CXCR3 chemokines are expressed in the pancreas, they may not contribute equally to CXCR3^+^ T_reg_ cell recruitment. The expression of CXCL9 by APCs and β-islet cells was much lower than CXCL10 and CXCL11. Considering CXCL9 is also the lowest affinity ligand and weakest inducer of migration of the three, it may prove less relevant to ICOS^+^ T_reg_ cell trafficking *in vivo*. Furthermore, as immune-endocrine interactions in hte islets shapediabetogenesis in NOD mice, we evaluated chemokine expression by islet cells during diabetes development. We found that T1D progression induced by T_eff_ cell transfer in NOD.TCRα^-/-^ hosts resulted in impaired CXCL9 and CXCL10 expression by insulin^+^ β-cells (ie live, non-destroyed) (Fig 5B and data not shown). Intriguingly, co-transfer of T_reg_ cells conferred dose-dependent protection from T_eff_ cell-mediated abrogation of chemokine production (Fig 5B). T_reg_-derived mediators could directly act on β-cells to induce chemokine expression, or enhance production of chemokines through protection of the islets from T_eff_ mediated cell death. Direct activation of apoptotic pathways by T_eff_-derived IFN-γ principally drives islet cell death in NOD mice, and indeed we found β cells expressed high levels of IFN-γR in our model compared to α and δ cells (Fig 5C) [26]. Furthermore, co-transfer of T_reg_ cells both reduced intra-pancreatic IFN-γ production by T_eff_ cells and protected β islet cells from cell death as measured by presence of insulin^+^ cells (S1C Fig). Thus, while T_eff_ cell transfer abolishes chemokine expression and drives autoimmunity, T_reg_ cells preserve CXCR3 ligand expression in the islets, perhaps through suppression of T_eff_ cell functions *in situ*. Overall, our findings indicate that APCs, T_reg_ cells and insulin-producing endocrine cells regulate CXCR3^+^ T_reg_ cell recruitment from the draining LN to Th1 inflamed pancreatic sites. These findings support the notion that insulin producing β-cells play an active role in modulating T_reg_ cell function in pancreatic sites.

### IFN-γ regulates CXCR3 expression and homing of ICOS^+^ T_reg_ cells to islets

**Fig 6:**
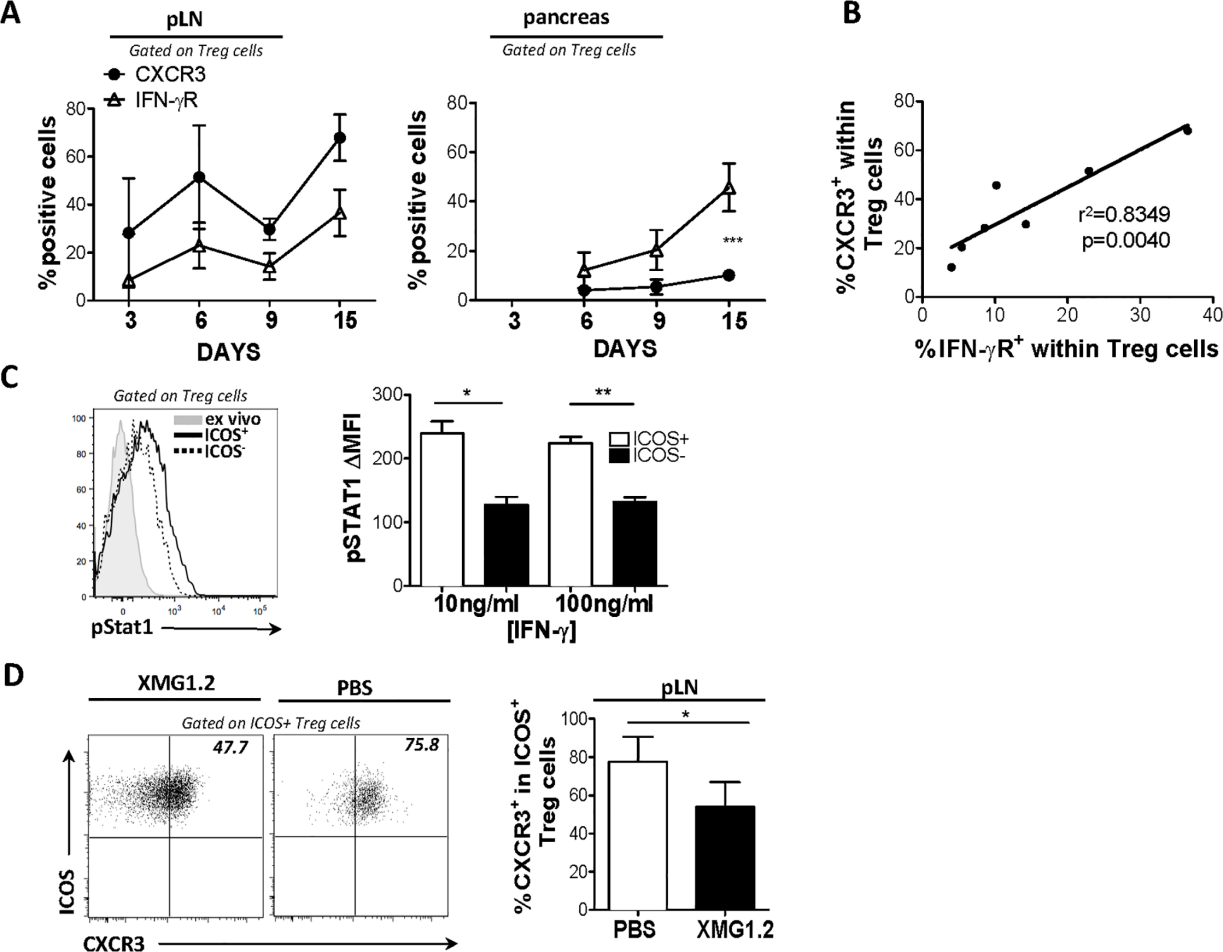
IFN-γ regulates CXCR3 expression by ICOS^+^ T_reg_ cells and their homing to islets. NOD.TCRα^**-/-**^ mice received BDC2.5 CD4^**+**^ T cells (7.5X10^**5**^) and, (A) and the percent CXCR3^**+**^ and IFN-γR^**+**^ cells among total T_**reg**_ cells in pancreas and draining LNs was assessed at the indicated times post-transfer. (B) Correlation between percent CXCR3^**+**^ and IFN-γR^**+**^ in pancreas and draining LN at all points examined. (C) BDC2.5 CD4^**+**^ T cells were stimulated with various concentrations of recombinant IFN-γ, and STAT1 phosphorylation was assessed by flow cytometry and compared between ICOS^**+**^ and ICOS^**-**^ subsets of T_**reg**_ cells. (D) NOD.TCRα^**-/-**^ mice received T_**eff**_ (7.5X10^**5**^) cells and were injected i.p. with either PBS or anti-IFNγ Ab (XMG1.2) on days -1, 1, 3, 5 and 7 post transfer. Mice were sacrificed when the PBS group displayed hyperglycemia (>33mmol/L). Cell suspensions of the pancreatic draining LN were obtained and the percent CXCR3^**+**^ among pT_**reg**_ cells was compared between groups. (6A, 6B, 6D n = 5. 6C n = 2).

We further investigated a role for IFN-γ in the upregulation of CXCR3 on T_reg_ cells. We assessed the expression of IFN-γ receptor (R) by CXCR3^+^ T_reg_ cells and found that CXCR3^+^ T_reg_ cells expressed higher levels of IFN-γR than CXCR3^-^ T_reg_ cells, suggesting that T_reg_ cells that express CXCR3 are more responsive to IFN-γ signaling. To elucidate the association between IFN-γR and CXCR3 expression over the course of T1D development, we examined the co-expression of these markers on donor CD4^+^ T cells following transfer into NOD.TCRα^-/-^ recipients. Mice were sacrificed on days 3, 6, 9 and 15 and CXCR3, IFN-γR and IFN-γ expression was assessed by flow cytometry on cell suspensions from pLN and pancreas. IFN-γ expression by pancreatic T_eff_ cells increased with T1D progression and peaked at day 9 (data not shown). CXCR3 and IFN-γR expression by ICOS^+^ T_reg_ cells correlated during the time course in all compartments evaluated (Fig 6A and 6B). Overall, this suggests increased IFN-γR signaling may selectively stimulate ICOS^+^ T_reg_ cells to upregulate CXCR3.

As CXCR3 expression was largely confined to ICOS^+^ T_reg_ cells, we hypothesized that a preferential responsiveness to IFN-γ resulted in upregulation of CXCR3 by this subset. Accordingly, we stimulated T cells isolated from pooled LN and spleen with recombinant IFN-γ, and examined the activity of IFN-γ signaling by assessing STAT1 activation *in vitro* using flow cytometry. ICOS^+^ T_reg_ cells, not only expressed higher levels of IFN-γR, but demonstrated higher STAT1 phosphorylation upon stimulation, indicating a more sensitive and activated IFN-γR signaling pathway than their ICOS^-^ counterparts, (Fig 6C). Overall, these results suggested to us that IFN-γ played a role in selective upregulation of CXCR3 by ICOS^+^ T_reg_ cells due to their enhanced sensitivity to this cytokine.

We next sought to confirm that IFN-γ regulated CXCR3 expression on ICOS^+^ T_reg_ *in vivo*. We predicted that anti-IFN-γ mAb treatment would impair expression of CXCR3 by ICOS^+^ T_reg_ cells upon transfer into NOD.TCRα^-/-^ recipients. To achieve this, we specifically neutralized IFN-γ activity in our transfer system using an anti-IFN-γ monoclonal antibody (mAb, XMG1.2), which potently inhibits IFN-γ *in vivo*. Our results show that anti-IFN-γ mAb treatment significantly blunted CXCR3 upregulation by ICOS^+^ pT_reg_ cells (Fig 6D) indicating that IFN-γ positively regulates expression of CXCR3 by ICOS^+^ T_reg_ cells during diabetogenesis. Thus, these results indicate that ICOS^+^ T_reg_ cells detect intra-islet IFN-γ produced by T_eff_ cells during T1D progression, and upregulate CXCR3 to home to the pancreas.

## Discussion

Chemokines and chemokine receptors are critical regulators of leukocyte trafficking during the immune response. Selective use of chemokine receptors by T_reg_ cells enables their homing to specific target tissues where they deploy their effector mechanisms to control inflammatory responses, as evidenced in models of infection and cancer. We previously demonstrated that during chronic *Leishmania major* infection, T_reg_ cells preferentially express CCR5 and home to cutaneous lesions in which they suppress T_eff_ cells and favor pathogen persistence [22]. Similarly, in human chronic liver disease and mouse models of hepatitis, CXCR3^+^ T_reg_ cells accumulate in the liver, where they dampen immune-mediated tissue damage [23, 24]. Further, Koch *et al*., demonstrated that during *Mycobacterium tuberculosis* infection, CXCR3^+^ T_reg_ cells accumulate and limit immune responses in the mediastinal LN [12]. Likewise, CXCR3^+^ T_reg_ cells were isolated from human ovarian carcinomas and shown to suppress T_eff_ proliferation and IFN-γ production *ex vivo* [25].

Studies of T_reg_ cell homing in NOD mice have implicated CXCR3 therein. Indeed, Yamada [26] and colleagues observed accelerated onset of T1D, and decrease pancreatic accumulation of T_reg_ cells in Cxcr3-/- NOD mice. A more recent study in NOD proinsulin 2-/- mice indicated that T_reg_ cells upregulate CXCR3 in the pLN and accumulate in the pancreas [27]. The results described here go further in defining a subpopulation of potently suppressive ICOS+ T_reg_ cells, which expresses CXCR3, the factors and cell types that may regulate T_reg_ cell CXCR3 expression and T_reg_ cell migration to the islets, and the functional and phenotypic characteristics of CXCR3-expressing T_reg_ cells. Specifically, this is the first demonstration, to our knowledge, of ICOS-dependent T_reg_ cell upregulation of trafficking receptors. However, these key findings were also corroborated in the non-TCR transgenic NOD mice, indicating that our proposed model may represent a mechanism of CXCR3 homing of T_reg_ cells to inflamed, non-lymphoid tissues that is conserved across strains (S1D Fig). Finally, this study makes use of a novel, multi-parametric flow cytometry approach for the assessment of endocrine islet cell function and thereby identifies a previously unrecognized role for β-cells in modulating T_reg_ cell responses

Diabetes induction by islet-reactive T_eff_ cells severely inhibited chemokine production by β-islet cells; however, their active insulin-secreting status suggests that the loss of chemokine expression by the islet cells is not merely a consequence of their destruction by T_eff_ cells alone. Strikingly, infusion of T_reg_ cells reverses T_eff_ cell-mediated abrogation of chemokine expression by ß-islet cells. This effect could be the result of suppression of intra-islet T_eff_ production of inflammatory cytokines by T_reg_ cells. For instance, T_eff_-derived IFN-γ can directly promote the apoptosis of islet cells as *stat1* ^*-/-*^ islets are resistant to BDC2.5 T_eff_-mediated killing *in vivo* [28]. Consistently, our results show that insulin^+^ ß-islet cells express IFN-γR indicating their capacity to respond to IFN-γ in our model. Thus, T_reg_ cell suppression of IFN-γ production *in situ* may both prevent T_eff_-mediated destruction of the islets and maintains β-islet production of CXCR3 chemokines that act to recruit T_reg_ cells. Intruigingly, the sole presence of T_reg_ cells in recipient mice augments islet production of CXCL10 suggesting that a direct T_reg_-islet cell interaction cannot be excluded.

An increase in Th1-mediated pancreatic inflammation leads to a dose-dependent upregulation of CXCR3 expression on T_reg_ cells, suggesting that T_eff_ cell-related signals or products, such as IFN-γ, induce mechanisms of pancreatic homing in T_reg_ cells. In other models, CXCR3^+^ T_reg_ cells co-express Foxp3 along with the IFN-γ-inducible Th1 lineage specifying transcription factor T-bet, which directly activates transcription of *Cxcr3* in addition to promoting responsiveness to IFN-γ through induction of IFN-γR expression [12]. This indicated that the IFN-γ-T-bet pathway is the basis of the robust upregulation of CXCR3 by ICOS^+^ T_reg_ cells. T-bet expression by ICOS^+^ T_reg_ cells correlated with CXCR3 expression *ex vivo* throughout T1D progression. Furthermore, the ICOS-expressing subset of T_reg_ cells preferentially expressed IFN-γ and IFN-γR, and displayed prominent STAT1 activation in response to IFN-γ *in vitro*. Moreover, neutralization of IFN-γ significantly blunted CXCR3 expression on ICOS^+^ T_reg_ cells in response to activation of islet-reactive T_eff_ cells *in vivo*. Taken as a whole, our results evince a model of ICOS^+^ T_reg_ cell homing to the islets in which IFN-γ acts directly on Foxp3+ Treg cells, and through IFN-γR and STAT1 signalling induces T-bet expression and CXCR3 upregulation. Equipped with CXCR3, T_reg_ cells then home towards inflamed islets in which immune and insulin-producing endocrine cells express CXCL9, CXCL10 and CXCL11.

While the expression of CXCR3 has been previously described in T_eff_ cells in murine models and patients with T1D, this is the first demonstration, to our knowledge, of ICOS-regulated CXCR3 expression by Foxp3^+^ T_reg_ cells [17, 31, 32]. In support of this, we show that CXCR3 and T-bet expression by T_reg_ cells is significantly diminished in ICOS^-/-^ mice. However, ICOS signaling has not been shown to induce T-bet expression by CD4^+^ T cells. Indeed, ICOS is well known to promote Th2 responses and more recently T-follicular helper cell differentiation, but shown to be unnecessary for Th1 differentiation [33]. However, the factors that govern T-bet expression in T_reg_ cells and Th1 cells may differ. Indeed, IL-12Rβ2 expression, which is indispensible for stable expression of T-bet by Th1 cells, was found to be abrogated during differentiation of CXCR3^+^ T_reg_ cells [30]. Therefore, in conjunction with IFN-γ signaling, induction of T-bet in T_reg_ cells may require additional stimuli involving ICOS. Previously, we showed that ICOS signals endow T_reg_ cells with an enhanced responsiveness to IL-2. Moreover, IL-2 can induce expression of T-bet in human CD4^+^ cells *in vitro* [34]. Therefore, responsiveness to IL-2 could potentially account for differential expression of T-bet between ICOS^+^ and ICOS^-^ T_reg_ cells.

In certain settings such as inflammation or lymphophenia, T_reg_ cells can lose Foxp3 expression, fully reprogram into a T_eff_ cell lineage and contribute to inflammatory processes [29]. Alternatively, recent studies have described transient forms of functional plasticity in T_reg_ cells expressing T_eff_ cell-associated markers critical for their trafficking and the survival at sites of Th1, Th2 or Th17 mediated pathogenesis [30, 13, 14]. Here, we show that CXCR3^+^ ICOS^+^ T_reg_ cells, the predominant and most immunosuppressive T_reg_ cell subset in the pancreas, express Th1-associated markers including the T-bet transcription factor. Thus ICOS^+^ T_reg_ cells do not completely reprogram into T_eff_ cells but acquire some Th1-like features, which may facilitate their preferential tissue-trafficking and functional fitness. Overall, this indicates that IFN-γ may not solely play a diabetogenic role but could also support tolerogenic mechanisms in the pancreas through regulation of T_reg_ cell homing.

Tissue homing of leukocytes profoundly affects immunity, as spatio-temporal coordination of immune cell subsets can dictate the nature of immune response. As homing is increasingly recognized as critical for T_reg_ cell-mediated suppression, it may represent a novel approach to enhancing T_reg_ cell functions. Notably, CXCR3^+^T_reg_ cells were recently shown to indispensably suppress Th1 responses, demonstrating they could be important in type 1 immune-mediated disease [12]. Concordantly, we show in a T1D model that CXCR3 is predominantly expressed by ICOS^+^ T_reg_ cells, presenting an avenue for selective enhancement of this suppressive subset of T_reg_ cells whilst minimizing recruitment of less suppressive ICOS^-^ T_reg_ cells. Though a very low percentage of T_eff_ cells express CXCR3, their use of this receptor cannot be entirely excluded. Thus, further elucidation of the factors that specifically modulate ICOS^+^ T_reg_ cell trafficking is required. If these are amenable to therapeutic modulation, their enhancement could potently dampen inflammation and control autoimmunity.

## Supporting Information

S1 Fig Cell suspensions of pancreas and draining LN from 4 week old WT or ICOS^-/-^ BDC2.5 mice were obtained and the frequency and level (MFI) of CXCR3 expression relative to ICOS and Foxp3 expression was assessed (A). NOD.TCRα^-/-^ mice received BDC2.5 CD4^+^CD25^+^ (T_reg_) or CD4^+^CD25^-^ (T_eff_, 7.5X10^5^) cells alone or at the indicated T_reg_/T_eff_ cell ratios. When the T_eff_ cell recipient mice displayed hyperglycemia (>33mmol/L), mice were sacrificed, and expression of IFN-γ by T_eff_ cells in pancLN was examined (B). Cell suspensions of the pancreas from 4 week old BDC2.5NOD mice were prepared and examined for CXCR3-ligand expression among Foxp3^+^ and Foxp3^-^ T cells (C). NOD.TCRα^-/-^ mice were left intact or received T_reg_ or T_eff_ cells at the indicated T_reg/_T_eff_ cell ratios. When mice receiving T_eff_ cells alone became hyperglycemic (>33mmol/L), insulin expression in β-islet cells was compared between groups (% insulin refers to % insulin^+^ cells out of total islet cells). (D). Cell suspensions from draining pLN of 4 week old BDC2.5 and NOD mice were obtained and the level of CXCR3 expression (MFI) between the ICOS^+^ and ICOS^-^ subsets of Foxp3+ T_reg_ cells was assessed. CXCR3 expression on Foxp3+ T_reg_ cells from BDC2.5 ICOS^-/-^ or NOD ICOS^-/-^ mice are also shown (E). Cell suspensions of draining LN from 4 week old BDC2.5 mice were obtained and assessed for T-bet expression (MFI). A representative plot showing the T-bet antibody stain relative to FMO control (on left) are shown. A T-bet antibody titration was performed using the indicated concentrations of antibody (right panel) (F).
(TIFF)
