# Supplementary figures and images for: Th1-Like ICOS+ Foxp3+ Treg Cells Preferentially Express CXCR3 and Home to β-Islets during Pre-Diabetes in BDC2.5 NOD Mice
Source: PLoS One. 2015 May 6;10(5):e0126311. doi: 10.1371/journal.pone.0126311 (PMC4422433; doi:10.1371/journal.pone.0126311)

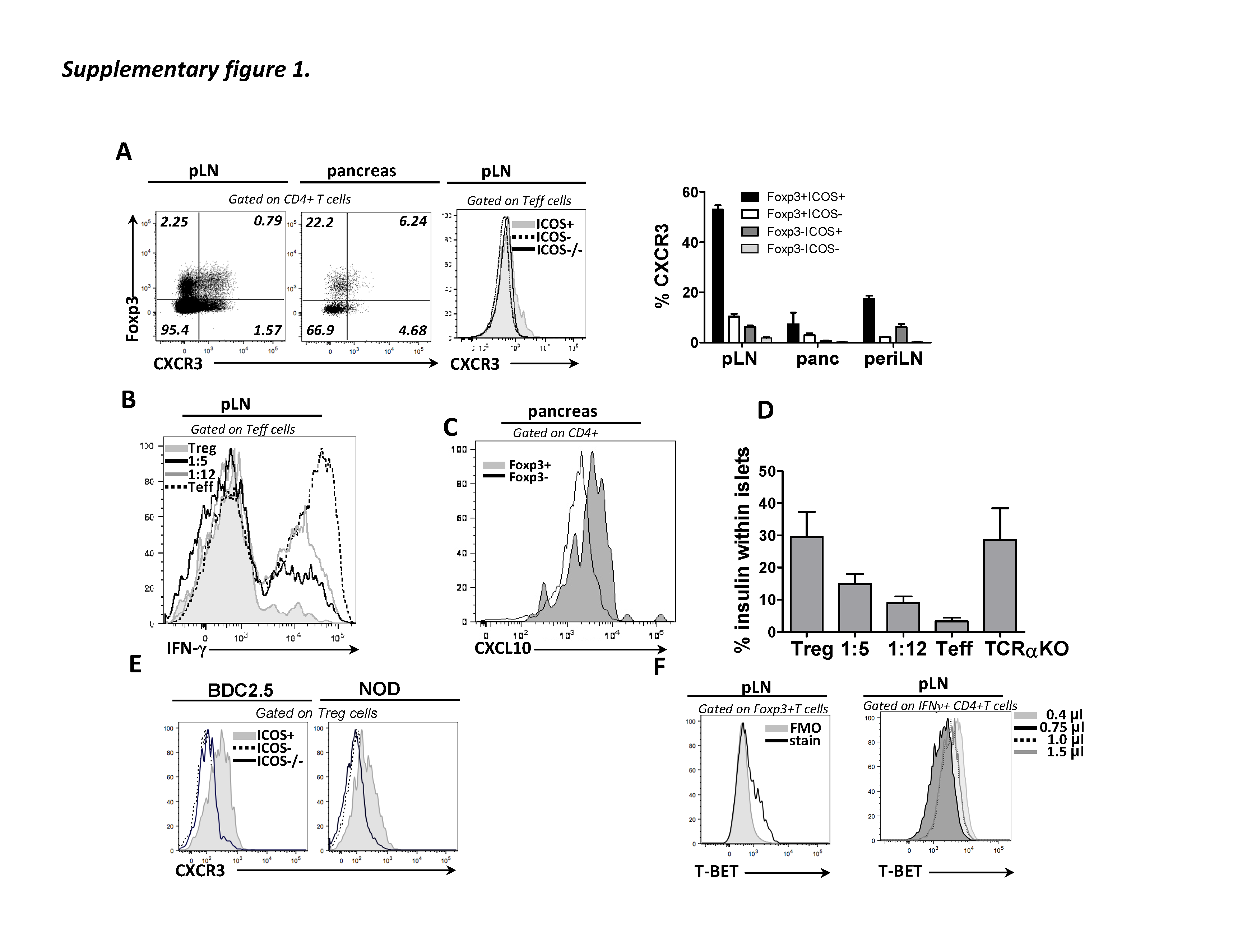

Supplement: S1 Fig — NOD.TCRα-/- mice received BDC2.5 CD4+CD25+ (Treg) or CD4+CD25- (Teff, 7.5X105) cells alone or at the indicated Treg/Teff cell ratios. When the Teff cell recipient mice displayed hyperglycemia (>33mmol/L), mice were sacrificed, and expression of IFN-γ by Teff cells in pancLN was examined (B). Cell suspensions of the pancreas from 4 week old BDC2.5NOD mice were prepared and examined for CXCR3-ligand expression among Foxp3+ and Foxp3- T cells (C). NOD.TCRα-/- mice were left intact or received Treg or Teff cells at the indicated Treg/Teff cell ratios. When mice receiving Teff cells alone became hyperglycemic (>33mmol/L), insulin expression in β-islet cells was compared between groups (% insulin refers to % insulin+ cells out of total islet cells). (D). Cell suspensions from draining pLN of 4 week old BDC2.5 and NOD mice were obtained and the level of CXCR3 expression (MFI) between the ICOS+ and ICOS- subsets of Foxp3+ Treg cells was assessed. CXCR3 expression on Foxp3+ Treg cells from BDC2.5 ICOS-/- or NOD ICOS-/- mice are also shown (E). Cell suspensions of draining LN from 4 week old BDC2.5 mice were obtained and assessed for T-bet expression (MFI). A representative plot showing the T-bet antibody stain relative to FMO control (on left) are shown. A T-bet antibody titration was performed using the indicated concentrations of antibody (right panel) (F). (TIFF) [file pone.0126311.s001.tiff]
